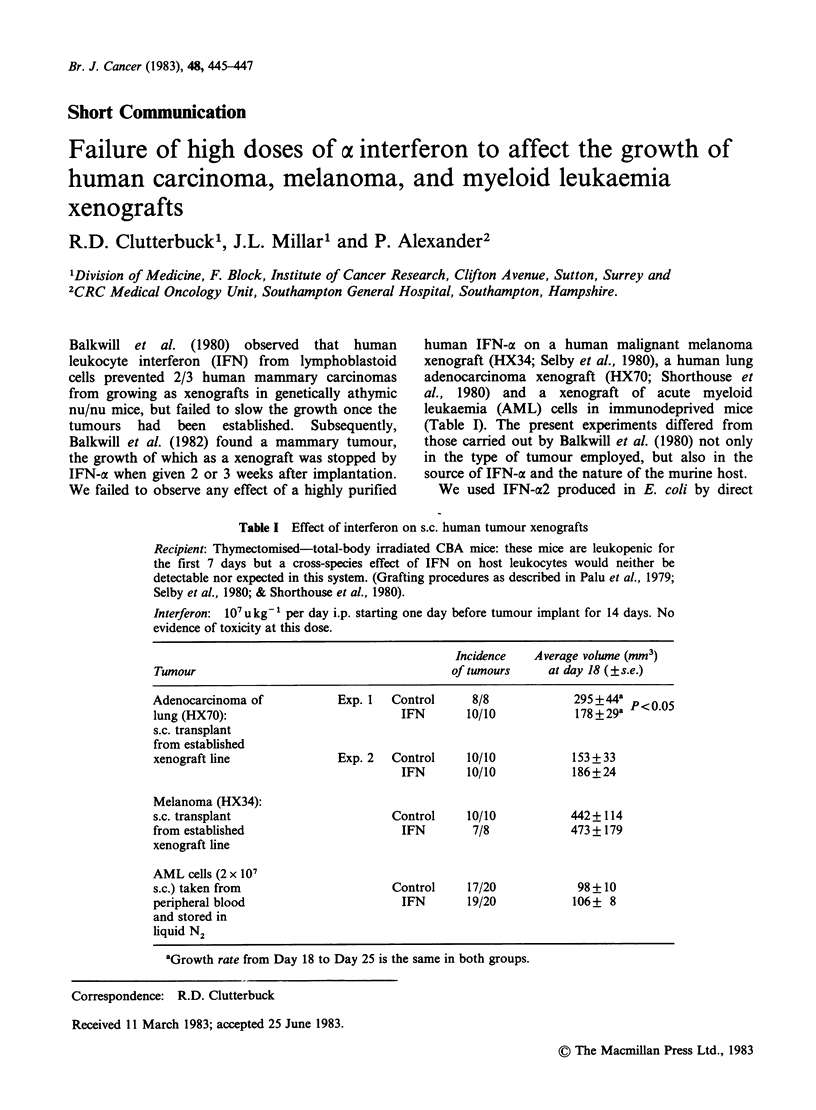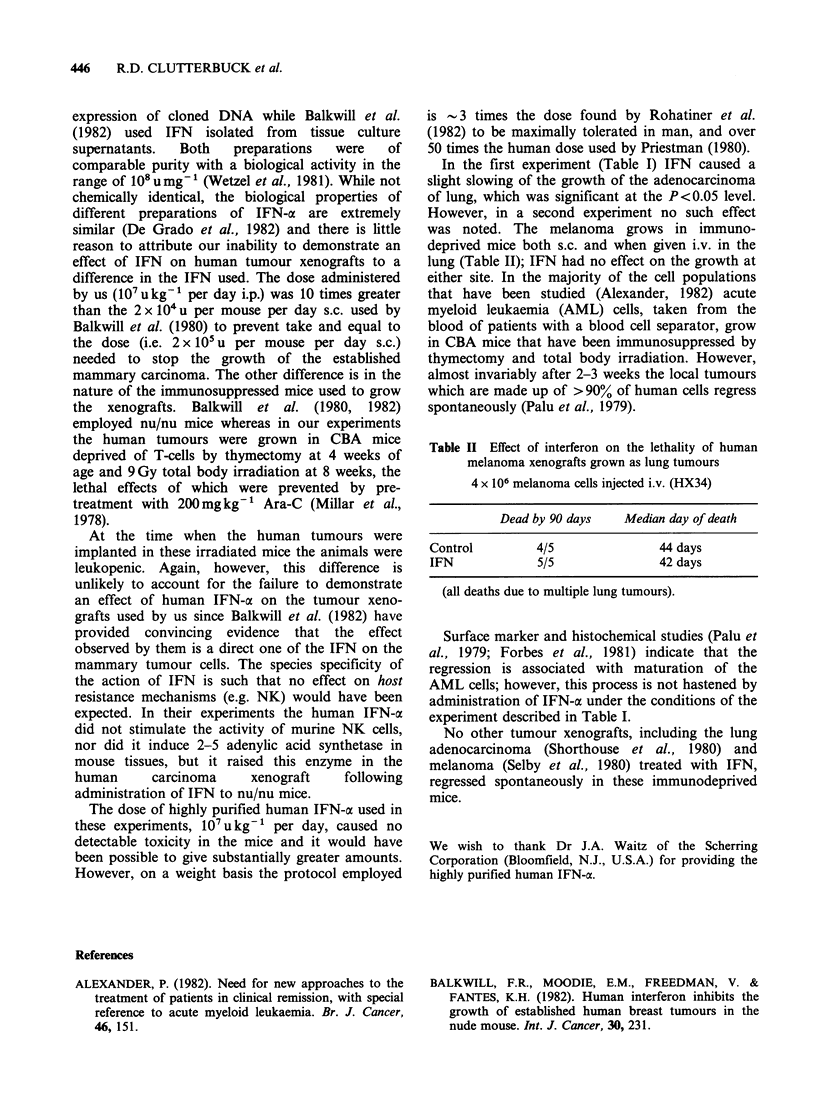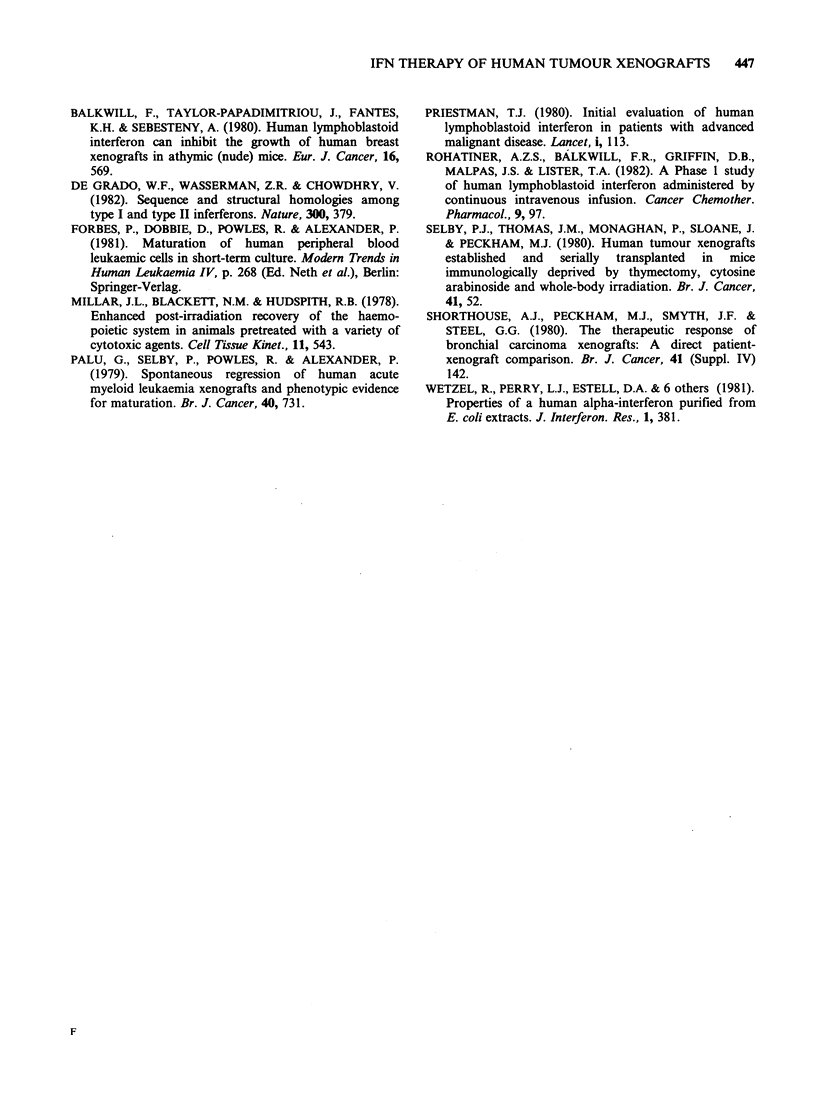# Failure of high doses of alpha interferon to affect the growth of human carcinoma, melanoma, and myeloid leukaemia xenografts.

**DOI:** 10.1038/bjc.1983.212

**Published:** 1983-09

**Authors:** R. D. Clutterbuck, J. L. Millar, P. Alexander


*Br. J. Cancer* (1983), **48**, 445–447

### Short Communication

### Failure of high doses of α interferon to affect the growth of human carcinoma, melanoma, and myeloid leukaemia xenografts

R.D. Clutterbuck[1], J.L. Millar[1] and P. Alexander[2]

[1]*Division of Medicine, F. Block, Institute of Cancer Research, Clifton Avenue, Sutton, Surrey and* [2]*CRC Medical Oncology Unit, Southampton General Hospital, Southampton, Hampshire.*

Balkwill *et al.* (1980) observed that human leukocyte interferon (IFN) from lymphoblastoid cells prevented 2/3 human mammary carcinomas from growing as xenografts in genetically athymic nu/nu mice, but failed to slow the growth once the tumours had been established. Subsequently, Balkwill *et al.* (1982) found a mammary tumour, the growth of which as a xenograft was stopped by IFN-α when given 2 or 3 weeks after implantation. We failed to observe any effect of a highly purified human IFN-α on a human malignant melanoma xenograft (HX34; Selby *et al.*, 1980), a human lung adenocarcinoma xenograft (HX70; Shorthouse *et al.*, 1980) and a xenograft of acute myeloid leukaemia (AML) cells in immunodeprived mice (Table I). The present experiments differed from those carried out by Balkwill *et al.* (1980) not only in the type of tumour employed, but also in the source of IFN-α and the nature of the murine host.

We used IFN-α2 produced in *E. coli* by direct

**Table I** Effect of interferon on s.c. human tumour xenografts

*Recipient*: Thymectomised—total-body irradiated CBA mice: these mice are leukopenic for the first 7 days but a cross-species effect of IFN on host leukocytes would neither be detectable nor expected in this system. (Grafting procedures as described in Palu *et al.*, 1979; Selby *et al.*, 1980; & Shorthouse *et al.*, 1980).

*Interferon*: $10^7 u kg^{-1}$ per day i.p. starting one day before tumour implant for 14 days. No evidence of toxicity at this dose.

| *Tumour* | | | *Incidence of tumours* | *Average volume (mm³) at day 18 (±s.e.)* | |
|---|---|---|---|---|---|
| Adenocarcinoma of lung (HX70): s.c. transplant from established xenograft line | Exp. 1 | Control | 8/8 | 295±44[a] | $P<0.05$ |
| | | IFN | 10/10 | 178±29[a] | |
| | Exp. 2 | Control | 10/10 | 153±33 | |
| | | IFN | 10/10 | 186±24 | |
| Melanoma (HX34): s.c. transplant from established xenograft line | | Control | 10/10 | 442±114 | |
| | | IFN | 7/8 | 473±179 | |
| AML cells ($2\times10^7$ s.c.) taken from peripheral blood and stored in liquid $N_2$ | | Control | 17/20 | 98±10 | |
| | | IFN | 19/20 | 106± 8 | |

[a]Growth *rate* from Day 18 to Day 25 is the same in both groups.

Correspondence: R.D. Clutterbuck

Received 11 March 1983; accepted 25 June 1983.

© The Macmillan Press Ltd., 1983

expression of cloned DNA while Balkwill *et al.* (1982) used IFN isolated from tissue culture supernatants. Both preparations were of comparable purity with a biological activity in the range of $10^8$ u mg$^{-1}$ (Wetzel *et al.*, 1981). While not chemically identical, the biological properties of different preparations of IFN-α are extremely similar (De Grado *et al.*, 1982) and there is little reason to attribute our inability to demonstrate an effect of IFN on human tumour xenografts to a difference in the IFN used. The dose administered by us ($10^7$ u kg$^{-1}$ per day i.p.) was 10 times greater than the $2 \times 10^4$ u per mouse per day s.c. used by Balkwill *et al.* (1980) to prevent take and equal to the dose (i.e. $2 \times 10^5$ u per mouse per day s.c.) needed to stop the growth of the established mammary carcinoma. The other difference is in the nature of the immunosuppressed mice used to grow the xenografts. Balkwill *et al.* (1980, 1982) employed nu/nu mice whereas in our experiments the human tumours were grown in CBA mice deprived of T-cells by thymectomy at 4 weeks of age and 9 Gy total body irradiation at 8 weeks, the lethal effects of which were prevented by pretreatment with 200 mg kg$^{-1}$ Ara-C (Millar *et al.*, 1978).

At the time when the human tumours were implanted in these irradiated mice the animals were leukopenic. Again, however, this difference is unlikely to account for the failure to demonstrate an effect of human IFN-α on the tumour xenografts used by us since Balkwill *et al.* (1982) have provided convincing evidence that the effect observed by them is a direct one of the IFN on the mammary tumour cells. The species specificity of the action of IFN is such that no effect on *host* resistance mechanisms (e.g. NK) would have been expected. In their experiments the human IFN-α did not stimulate the activity of murine NK cells, nor did it induce 2–5 adenylic acid synthetase in mouse tissues, but it raised this enzyme in the human carcinoma xenograft following administration of IFN to nu/nu mice.

The dose of highly purified human IFN-α used in these experiments, $10^7$ u kg$^{-1}$ per day, caused no detectable toxicity in the mice and it would have been possible to give substantially greater amounts. However, on a weight basis the protocol employed is ~3 times the dose found by Rohatiner *et al.* (1982) to be maximally tolerated in man, and over 50 times the human dose used by Priestman (1980).

In the first experiment (Table I) IFN caused a slight slowing of the growth of the adenocarcinoma of lung, which was significant at the $P < 0.05$ level. However, in a second experiment no such effect was noted. The melanoma grows in immunodeprived mice both s.c. and when given i.v. in the lung (Table II); IFN had no effect on the growth at either site. In the majority of the cell populations that have been studied (Alexander, 1982) acute myeloid leukaemia (AML) cells, taken from the blood of patients with a blood cell separator, grow in CBA mice that have been immunosuppressed by thymectomy and total body irradiation. However, almost invariably after 2–3 weeks the local tumours which are made up of >90% of human cells regress spontaneously (Palu *et al.*, 1979).

**Table II** Effect of interferon on the lethality of human melanoma xenografts grown as lung tumours

$4 \times 10^6$ melanoma cells injected i.v. (HX34)

| | *Dead by 90 days* | *Median day of death* |
|---|---|---|
| Control | 4/5 | 44 days |
| IFN | 5/5 | 42 days |

(all deaths due to multiple lung tumours).

Surface marker and histochemical studies (Palu *et al.*, 1979; Forbes *et al.*, 1981) indicate that the regression is associated with maturation of the AML cells; however, this process is not hastened by administration of IFN-α under the conditions of the experiment described in Table I.

No other tumour xenografts, including the lung adenocarcinoma (Shorthouse *et al.*, 1980) and melanoma (Selby *et al.*, 1980) treated with IFN, regressed spontaneously in these immunodeprived mice.

We wish to thank Dr J.A. Waitz of the Scherring Corporation (Bloomfield, N.J., U.S.A.) for providing the highly purified human IFN-α.

### References

ALEXANDER, P. (1982). Need for new approaches to the treatment of patients in clinical remission, with special reference to acute myeloid leukaemia. *Br. J. Cancer*, **46**, 151.

BALKWILL, F.R., MOODIE, E.M., FREEDMAN, V. & FANTES, K.H. (1982). Human interferon inhibits the growth of established human breast tumours in the nude mouse. *Int. J. Cancer*, **30**, 231.

BALKWILL, F., TAYLOR-PAPADIMITRIOU, J., FANTES, K.H. & SEBESTENY, A. (1980). Human lymphoblastoid interferon can inhibit the growth of human breast xenografts in athymic (nude) mice. *Eur. J. Cancer*, **16**, 569.

DE GRADO, W.F., WASSERMAN, Z.R. & CHOWDHRY, V. (1982). Sequence and structural homologies among type I and type II inferferons. *Nature*, **300**, 379.

FORBES, P., DOBBIE, D., POWLES, R. & ALEXANDER, P. (1981). Maturation of human peripheral blood leukaemic cells in short-term culture. *Modern Trends in Human Leukaemia IV*, p. 268 (Ed. Neth *et al.*), Berlin: Springer-Verlag.

MILLAR, J.L., BLACKETT, N.M. & HUDSPITH, R.B. (1978). Enhanced post-irradiation recovery of the haemopoietic system in animals pretreated with a variety of cytotoxic agents. *Cell Tissue Kinet.*, **11**, 543.

PALU, G., SELBY, P., POWLES, R. & ALEXANDER, P. (1979). Spontaneous regression of human acute myeloid leukaemia xenografts and phenotypic evidence for maturation. *Br. J. Cancer*, **40**, 731.

PRIESTMAN, T.J. (1980). Initial evaluation of human lymphoblastoid interferon in patients with advanced malignant disease. *Lancet*, **i**, 113.

ROHATINER, A.Z.S., BÁLKWILL, F.R., GRIFFIN, D.B., MALPAS, J.S. & LISTER, T.A. (1982). A Phase 1 study of human lymphoblastoid interferon administered by continuous intravenous infusion. *Cancer Chemother. Pharmacol.*, **9**, 97.

SELBY, P.J., THOMAS, J.M., MONAGHAN, P., SLOANE, J. & PECKHAM, M.J. (1980). Human tumour xenografts established and serially transplanted in mice immunologically deprived by thymectomy, cytosine arabinoside and whole-body irradiation. *Br. J. Cancer*, **41**, 52.

SHORTHOUSE, A.J., PECKHAM, M.J., SMYTH, J.F. & STEEL, G.G. (1980). The therapeutic response of bronchial carcinoma xenografts: A direct patient-xenograft comparison. *Br. J. Cancer*, **41** (Suppl. IV) 142.

WETZEL, R., PERRY, L.J., ESTELL, D.A. & 6 others (1981). Properties of a human alpha-interferon purified from *E. coli* extracts. *J. Interferon. Res.*, **1**, 381.